# Contest experience enhances aggressive behaviour in a fly: when losers learn to win

**DOI:** 10.1038/srep09347

**Published:** 2015-03-20

**Authors:** Giovanni Benelli, Nicolas Desneux, Donato Romano, Giuseppe Conte, Russell H. Messing, Angelo Canale

**Affiliations:** 1 Department of Agriculture, Insect Behaviour Group, Food and Environment, University of Pisa, via del Borghetto 80, 56124 Pisa, Italy; 2 French National Institute for Agricultural Research (INRA), Sophia-Antipolis, France; 3 Kauai Agricultural Research Center, University of Hawaii at Manoa, 7370 Kuamo'o Road, Kapaa, Hawaii 97646 USA

**Keywords:** Behavioural ecology, Entomology

## Abstract

In several animal species, aggressive experience influences the characteristics and outcomes of subsequent conflicts, such that winners are more likely to win again (the winner effect) and losers more likely to lose again (the loser effect). We tested the olive fruit fly, *Bactrocera oleae* (Diptera: Tephritidae), as a model system to evaluate the role of the winner and loser effects in male-male territorial contests. Further, we conducted experiments to test if winning and losing probabilities are affected only by the outcome of the previous contests, or whether the fighting experience itself is sufficient to induce an effect. Both winners and losers of two consecutive encounters displayed higher intensity of aggression and fought longer in subsequent contests. In both cases, they achieved higher fighting success than naïve males. The enhanced fighting performance of both winners and losers was stimulated by merely experiencing a contest, not necessarily by the relative outcome of previous fights. Overall, this study highlights the fact that previous victories and defeats both enhance aggressive behaviour in olive fruit flies, allowing them to achieve higher fighting success in subsequent contests against inexperienced males.

## Introduction

Aggressive behaviour is widespread throughout the animal kingdom^1,2^. Aggression is a highly flexible behaviour affected by a large number of factors and is important to ensure survival and reproduction in many species^3^. The evolution of aggression is shaped by a trade-off between the resultant benefits (i.e., securing limited resources) vs. costs (i.e., risk of injury; loss of time and energy), optimising fitness outcomes^4,5^. Aggressive behaviour can be severe between individuals of the same species, as they compete for the same food, territory and access to mates^6^. Game theory predicts that Evolutionarily Stable Strategies for conflicts between conspecifics may involve stereotyped contests characterized by the ritualised exchange of agonistic signals^7^, which are thought to convey increasingly accurate information for assessing the contenders' chances of winning^8,9,10,11^. Probability of winning can depend on physical disparities (e.g. size, strength, weaponry)^12,13,14,15^ as well as on aggressive motivation^16^. The latter is a product of several factors, including the presence of resources^17,18,19,20,21^, social upbringing^22^, physical exertion^23^ and experience in previous fights^24,25,26^.

Learning in the context of aggressive behaviour is widely recognised in animals^4,27,28^ and it has been shown that previous contest experience affects the characteristics and outcomes of contests in many species^4,26^. It is generally acknowledged that behavioural changes during combat that relate to prior experience fall into two general categories. Losing experiences tend to decrease willingness to engage in a contest (i.e. the loser effect), while winning experiences tend to increase willingness to escalate a contest (i.e. the winner effect)^4^. In other words, individuals experiencing a previous victory escalate to higher levels of aggression and fight longer in subsequent contests^27,28^, while losers tend to avoid further contests and show reduced aggression^4,29,30,31^. It has been proposed that the winner and the loser effects can result from a reassessment by contestants of their perceived fighting abilities^4,32^. Game-theory models based on this assumption predict that the loser effect can exist alone or in the presence of a winner effect, while the winner effect cannot persist alone, at least when contestants are young and without fighting experience^32,33,34,35^. Furthermore, when both effects coexist, the loser effect is predicted to be longer and of greater magnitude than the winner effect^4,36^. However, a recent study by Goubault and Décuignere^37^ showed for the first time that the winner effect exists in the absence of any evident loser effect in the parasitic wasp *Eupelmus vuilleti* (Craw), when females fight for hosts. To explain the evolution of independent winner effects, an alternative hypothesis has been proposed based on modification of the contestants' subjective valuation of the resource, rather than on a re-estimation of their fighting abilities^37^.

Several neuroendocrine mechanisms mediating experience effects on aggressive behaviour have been outlined. In vertebrates (e.g., fish and mice), the winner effect seems to be mediated by androgens^38,39^, while in invertebrates (e.g., crickets) it has been shown that the winner effect is modulated via a mechanism involving release of the biogenic amine octopamine^25,40^. Evidence about proximate mechanisms guiding the loser effect is patchier and varies among different species^4^. Among vertebrates, elevated levels of corticosteroids (i.e., pituitary-adrenocortical hormones that increase in titre during stress) are often detected in losers. In several vertebrate species depressed plasma testosterone levels accompany defeat and lower 11-ketotestosterone levels have also been observed^4,36,41^. In invertebrates, serotonin (5HT), nitric oxide (NO) and selected peptides affect the tendency to flee, avoiding further contests^10,42^.

To date, relatively few model systems have been developed to shed light on how previous experience modifies aggressive behaviour in arthropods, mainly crickets^26^, drosophilid flies^43^ and ants^28^ (see also recent reviews^4,9,22,44^). We propose a new model system, the olive fruit fly, *Bactrocera oleae* (Rossi) (Diptera: Tephritidae). *B. oleae* is a worldwide olive fruit pest of great economic importance^45,46^. Aggressive behaviour is important in this species. During late afternoon, *B. oleae* males form swarms on the windward side of olive trees. Within a swarm, each male fights to defend a small territory (an olive leaf) where they court and mate female flies^21,46^. Olive fruit fly females also display agonistic behaviour to maintain single oviposition sites and reduce larval competition for food^21,45^. *B. oleae* aggressive behaviour is highly ritualized, composed of a number of distinct behavioural acts including synchronous wing waving, fast running towards the opponent, pouncing and boxing with forelegs^21,45^. Resident flies win more contests than intruders^21^, where a win is defined as retention of the favoured location, while a loss is the departure from that location. Olive fruit flies do not damage each other during fights^21^.

From a behavioural point of view, the winner and loser effects are thought to be the result of prior winning and losing experience influencing an individual's assessment of its own fighting ability and estimated costs of fighting in subsequent contests^4,47^, thus allowing a prompt “fight or flee” decision prior to escalation. This issue has been poorly studied in Tephritidae flies. We hypothesize that the winner effect plays a role guiding the aggressive behaviour in olive fruit fly males fighting for territories and mates (Experiment 1). Furthermore, in an associative learning context, experiencing a victory can be considered a reward, while a defeat is a “punishment” or aversive stimulus. On this basis, we supposed that losers gain information from contest experience, enhancing their fighting strategy in subsequent contests (Experiment 2). The acquisition of information from previous experience, regardless of outcome, is often exploited by animals to refine their future performance^3^, especially in courtship and mating activities^48,49^. Our last question was “what is the importance of prior physical combat in determining the fighting outcome?” To attempt a reply, we evaluated if winning and losing probabilities are affected solely by the outcome of the previous contests, or if they required actual experience in physical combat (Experiment 3).

## Results

### Experiment 1: the winner effect

**Figure 1:**
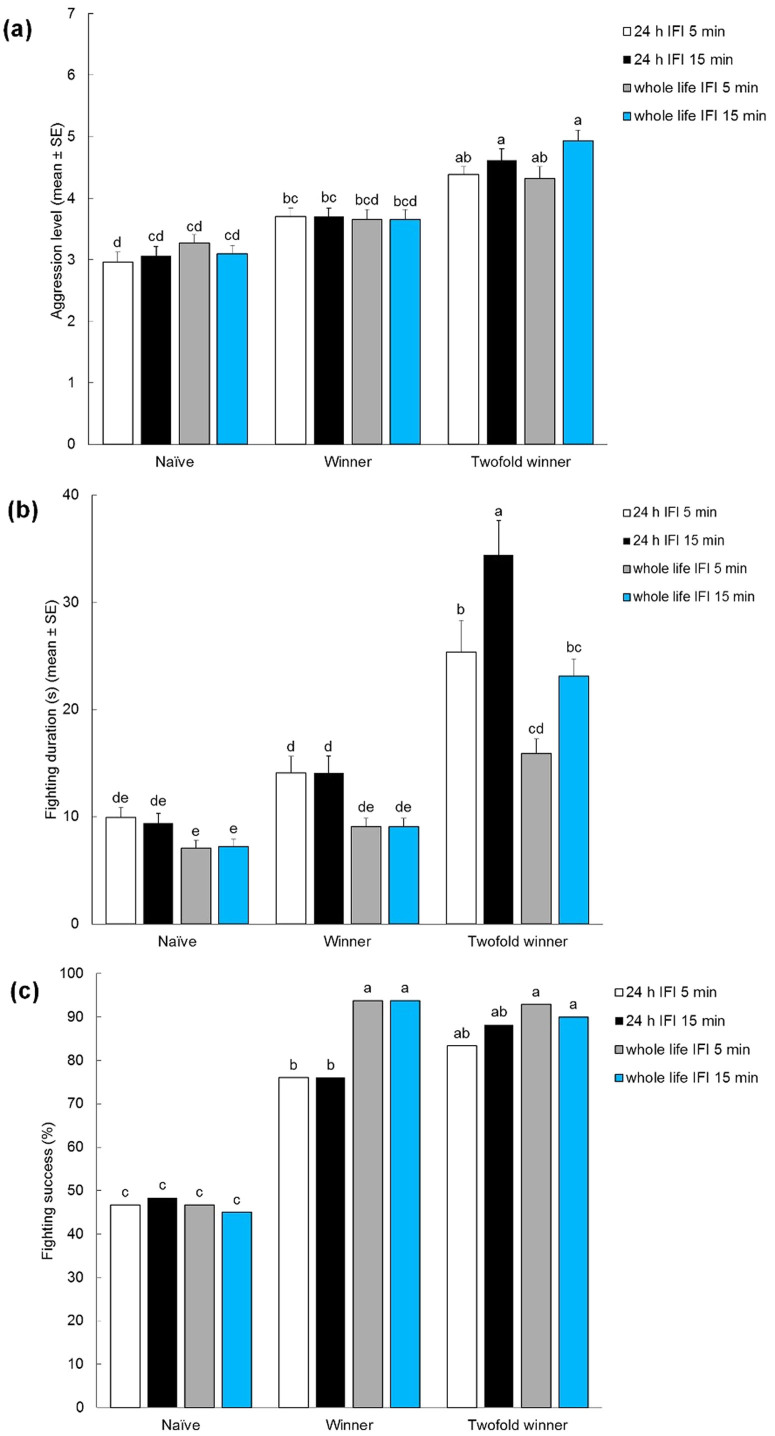
Bar graphs giving mean intensity (A), duration (B) and success (C) of male-male contests in olive fruit flies, *Bactrocera oleae*, with different winning experiences. Naïve = fight-inexperienced male. Winner = winner of one previous encounter. Twofold winner = winner of two previous encounters. Inter-fight intervals (IFI) were 5 min and 15 min. Both flies exposed to artificially crowded conditions until 24 h before the experimental phase and individuals isolated for their entire life until the experimental phase were tested. Different letters above each bar indicated significant differences. T-bars are standard errors.

In male-male contests, the intensity of aggression was significantly affected by experience (*F*_*2, 356*_ = 86.047, *P* < 0.001), while the effects of isolation, inter-fight interval and interactions between inter-fight interval*experience, isolation*experience, isolation*inter-fight interval and isolation*inter-fight interval*experience were not significant. Intensity of aggression was higher in males that previously won twice in succession (twofold winners) than in naïve males. Performances of twofold winners and males that previously won once (winners) were comparable (Figure 1a). Within each experience treatment, no differences were observed between males subjected to 24 h vs. lifetime pre-experimental isolation, nor were differences observed between aggression levels in contests after an inter-fight interval of 5 vs. 15 min (Figure 1a).

Fight duration was significantly influenced by experience (*F*_*2, 401*_ = 118.873, *P* < 0.001), isolation (*F*_*1, 231*_ = 47.764, *P* < 0.001), inter-fight interval (*F*_*1, 231*_ = 9.184, *P* = 0.003) and the interactions between inter-fight interval*experience (*F*_*2, 401*_ = 8.549, *P* < 0.001) and isolation*experience (*F*_*2, 401*_ = 8.995, *P* < 0.001), while the effect of interactions isolation*inter-fight interval and isolation*inter-fight interval*experience was not significant. Fight duration was longer in twofold winners than in naïve and winner males (Figure 1b). Within naïve and winner males, no differences were observed between males subjected to 24 h vs. lifetime pre-experimental isolation, nor were differences observed between fighting duration displayed after an inter-fight interval of 5 vs. 15 min. Within the twofold winner treatment, fighting duration was slightly longer in males subjected to 24 h pre-experimental isolation than in lifetime-isolated males (Figure 1b) and slightly longer in those with inter-fight interval of 15 min than those with inter-fight interval of 5 min (for 24 h isolated males only).

Fighting success was significantly affected by experience (*F*_*2, 792*_ = 247.709, *P* < 0.001), isolation (*F*_*1, 396*_ = 5.676, *P* = 0.018) and the interaction between isolation*experience (*F*_*2, 792*_ = 10.989, *P* < 0.001), while the effects of inter-fight interval and the interactions between isolation*inter-fight interval, inter-fight interval*experience, isolation*inter-fight interval*experience were not significant. Fighting success was lower in naïve males than in winners and twofold winners, regardless of pre-experimental isolation and inter-fight interval. No differences were detected between fighting success of winners and twofold winners (Figure 1c).

### Experiment 2: the loser effect

**Figure 2:**
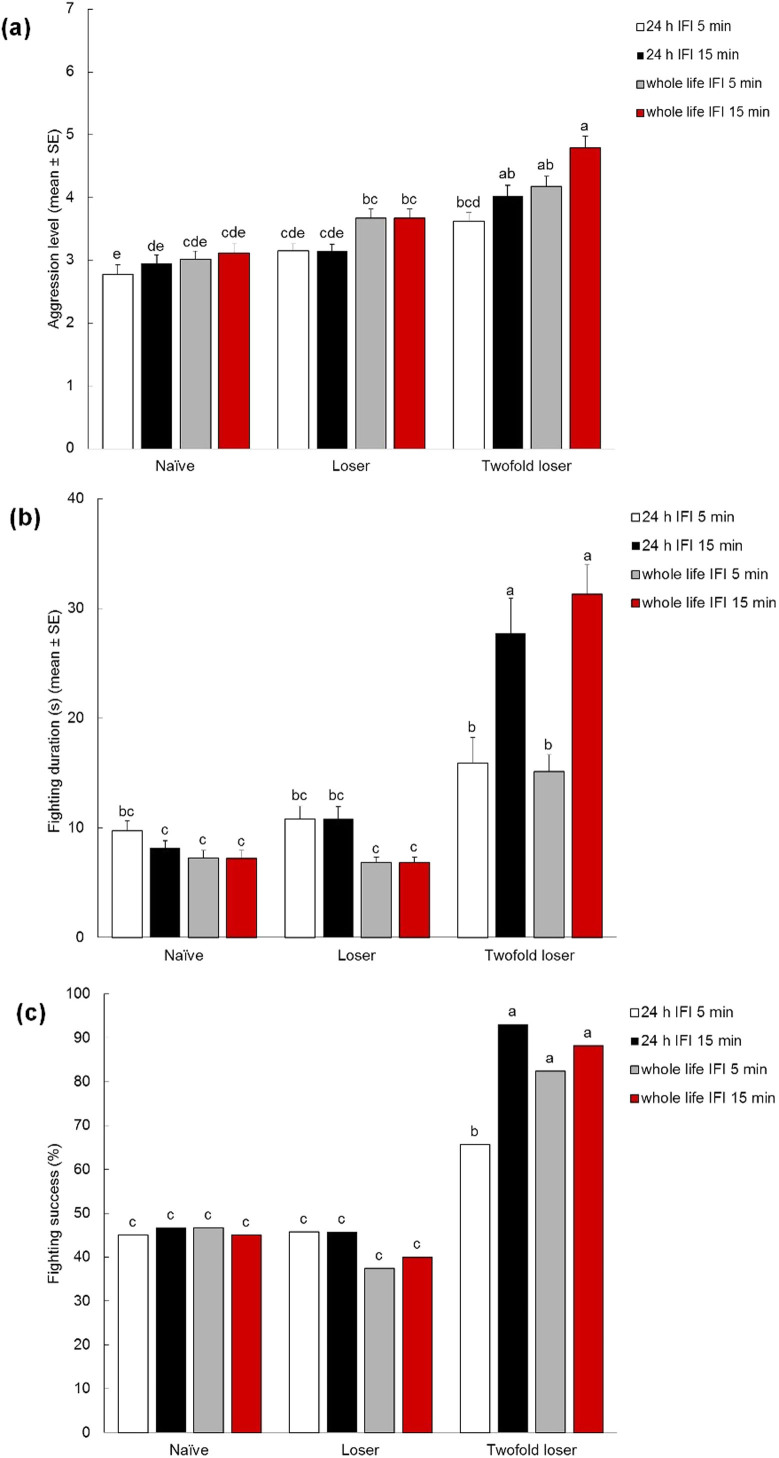
Bar graphs giving mean intensity (A), duration (B) and success (C) of male-male contests in olive fruit flies, *Bactrocera oleae*, with different losing experiences. Naïve = fight-inexperienced male. Loser = loser of one previous encounter. Twofold loser = loser of two previous encounters. Inter-fight intervals were (IFI) 5 min and 15 min. Both flies exposed to artificially crowded conditions until 24 h before the experimental phase and individuals isolated for their entire life until the experimental phase were tested. Different letters above each bar indicated significant differences. T-bars are standard errors.

In male-male contests, the intensity of aggression was significantly affected by experience (*F*_*2, 392*_ = 62.866, *P* < 0.001), isolation (*F*_*1, 216*_ = 28.417, *P* < 0.001) and inter-fight interval (*F*_*1, 216*_ = 6.268, *P* = 0.013), while the effects of the interactions between isolation*experience, inter-fight interval*experience, isolation*inter-fight interval and isolation*inter-fight interval*experience were not significant. The intensity of aggression was higher in males that had previously lost twice in succession (twofold losers) than the intensity in naïve males (Figure 2a). Within each experience treatment, no differences were observed between males subjected to 24 h vs. lifetime pre-experimental isolation; nor were differences observed between responses displayed after an inter-fight interval of 5 vs. 15 min. The only exception was that lifetime isolated twofold losers, tested with an inter-fight interval of 15 min, showed more intense aggression in contests than 24 h isolated twofold losers tested with an inter-fight interval of 5 min (Figure 2a).

Fighting duration was significantly affected by experience (*F*_*2, 410*_ = 130.644, *P* < 0.001), inter-fight interval (*F*_*1, 238*_ = 32.574, *P* < 0.001) and the interactions between inter-fight interval*experience (*F*_*2, 410*_ = 34.309, *P* < 0.001), isolation*experience (*F*_*2, 410*_ = 3.655, *P* = 0.027), while the effects of isolation and the interactions between isolation*inter-fight interval and isolation*inter-fight interval*experience were not significant. Fighting duration was longer in twofold losers than in naïve males and males that previously lost only once (Figure 2b). No differences were observed between naïve and losers. In naïve and loser treatments, fighting duration was comparable, regardless of pre-experimental isolation and inter-fight interval. Within the twofold loser treatment, no differences were found between males subjected to lifetime vs. 24 h isolation. In contrast, twofold losers fought for longer durations with an inter-fight interval of 15 min than with an inter-fight interval of 5 min (Figure 2b).

Fighting success was significantly affected by experience (*F*_*2, 792*_ = 237.157, *P* < 0.001) and the interactions between isolation*experience (*F*_*2, 792*_ = 4.804, *P* = 0.008) and inter-fight interval*experience (*F*_*2, 792*_ = 10.475, *P* < 0.001), while the effects of isolation, inter-fight interval and the interactions between isolation*inter-fight interval and isolation*inter-fight interval*experience were not significant. Fighting success was higher in twofold losers than in naïve and loser males. No consistent differences in fighting success were found between twofold losers subjected to different pre-experimental isolation periods, or inter-fight intervals. No differences in fighting success were found between naïve and loser males (Figure 2c).

### Experiment 3: role of physical combat in determining winner and loser effect

Here four categories of experienced males were tested against naïve males: (1) winners male that had won twice in succession with physical fighting (winners with fighting), (2) winner males that had won twice in succession without physical fighting (winners without fighting), (3) loser males that had lost twice in succession with physical fighting (losers with fighting); (4) loser males that lost twice in succession without physical fighting (losers without fighting).

**Figure 3:**
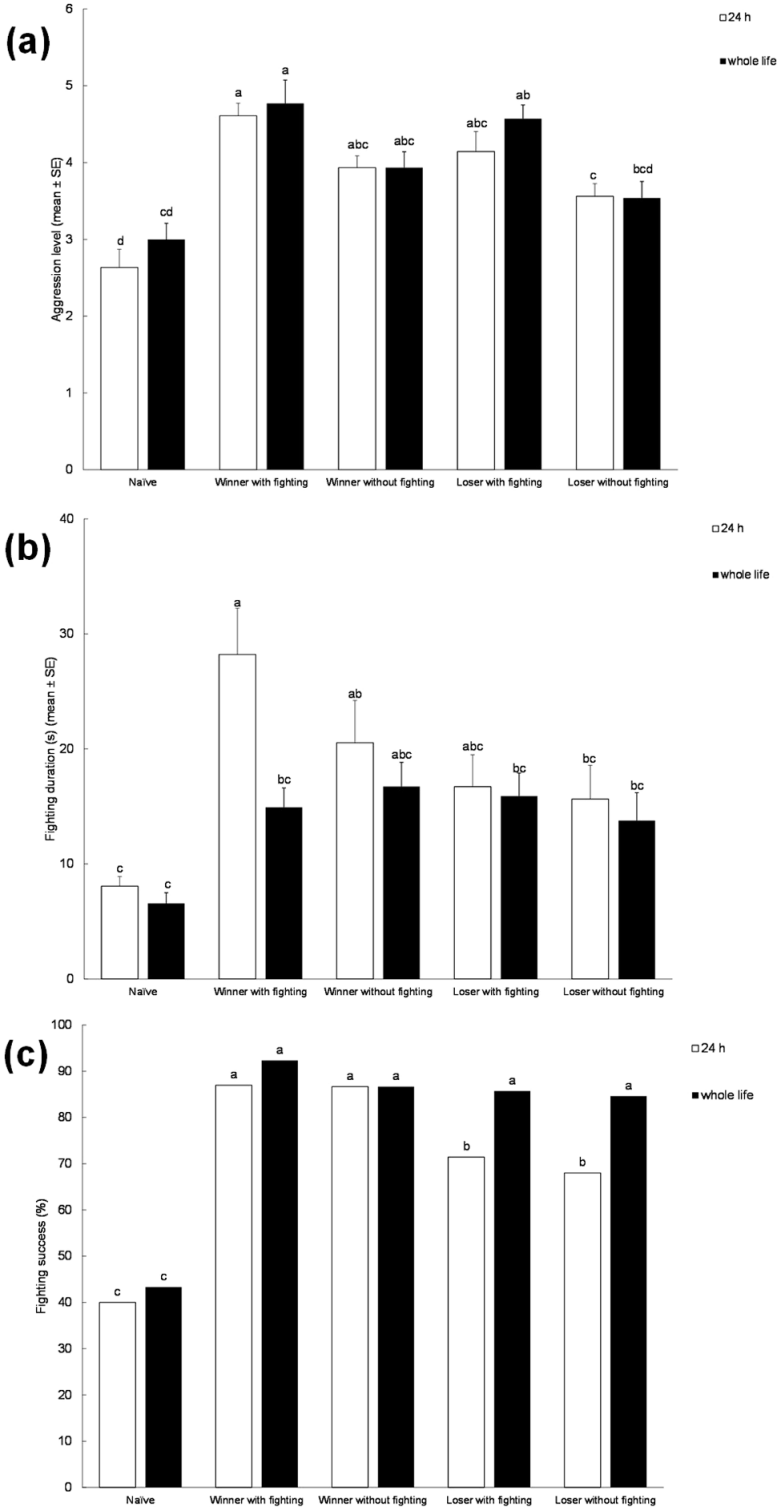
Bar graphs giving mean intensity (A), duration (B) and success (C) of male-male contests in olive fruit flies, *Bactrocera oleae*, with different winning and losing experiences: the role of fighting experience itself. Naïve = fight-inexperienced male. Winner with fighting = winner male that had won twice in succession with physical fighting. Winner without fighting = winner male that had won twice in succession without physical fighting. Loser with fighting = loser male that had lost twice in succession with physical fighting. Loser without fighting = loser male that lost twice in succession without physical fighting. Inter-fight intervals (IFI) were 5 min and 15 min. Both flies exposed to artificially crowded conditions until 24 h before the experimental phase and individuals isolated for their entire life until the experimental phase were tested. Different letters above each bar indicated significant differences. T-bars are standard errors.

The intensity of aggression was significantly influenced by experience (*F*_*4, 111*_ = 23.892, *P* < 0.001), while the effects of isolation and the interaction between isolation*experience were not significant. The intensity of aggression was comparable between winners and losers of two previous contests (Figure 3a). Both within winners and losers, no consistent differences were found between males who had experienced physical combat and those who had not. Naïve males had lower aggression levels than those males with experience, with the exception of losers without fighting, who had comparable levels of aggression as naïve males. Within all experience treatments, there were no differences between males subjected to lifetime vs. 24 h isolation (Figure 3a).

Fighting duration was significantly affected by experience (*F*_*4, 113*_ = 10.648, *P* < 0.001) and isolation (*F*_*1, 105*_ = 6.227, *P* = 0.014), while the interaction of isolation*experience was not significant. Fighting duration was lower in naïve males over the others (Figure 3b). Within all experience treatments, there were no differences between males subjected to lifetime vs 24 h isolation, with a single exception: among combat winners, males isolated for 24 h fought longer than those isolated for their entire lifetime (Figure 3b).

Fighting success was significantly affected by experience (*F*_*4, 990*_ = 111.659, *P* < 0.001) and isolation (*F*_*1, 990*_ = 10.093, *P* = 0.002) but not by the interaction between isolation*experience. Fighting success was lower in naïve males over the other males, regardless of the isolation period. No consistent differences in fighting success were detected among experienced males (Figure 3c).

## Discussion

Previous aggressive experience influences the outcome of conflicts, such that winners are more likely to win again and losers will more likely lose again^26,32,37,50^. In agreement with our first hypothesis, our data on the winner effect (Experiment 1) are consistent with earlier studies in other invertebrate species, in which aggression levels, fighting duration and/or probability of winning were higher in previous contest winners than in naïve ones (i.e. crickets^51^ and crayfish^52^). The winner effect is also widely recognised in a number of vertebrates, including fish (e.g. stickleback, pumpkinseed sunfish, mangrove rivulus, blue gourami), birds (e.g. blue-footed booby) and mammals (e.g. mice)^4^.

In our second hypothesis, we supposed that *B. oleae* losers are able to gain information from contest experience, enhancing their fighting strategy in subsequent contests. Data from Experiment 2 supported our prediction, showing that losers needed two previous defeats to display a higher intensity of aggression, to fight for longer durations and to achieve greater fighting success in subsequent contests than naïve males. The observed effect lasted at least 15 min. To the best of our knowledge, similar effects have rarely been observed, either in invertebrates^53^ or vertebrates^54,55^. Most studies on invertebrate species reported decreased intensity of aggression and/or lower fighting duration in individuals that experienced a defeat in previous contests, compared to naïve individuals or winners (e.g. crickets and *Drosophila* flies), using both self-selection and random selection procedures^4,24,27,30,51^. Similar findings have been reported for vertebrates, such as fish (e.g. stickleback, green sunfish, pumpkinseed sunfish, paradise fish and mangrove rivulus), reptiles (e.g. copperhead snake), birds (e.g. blue-footed booby) and mammals (e.g. mice)^4,56^. Why olive fruit flies show a different response to prior social experience compared to other invertebrate and vertebrate species is unclear. Further research is needed to understand if previous fighting experience can modify the contestants' subjective value of a given resource (i.e. territory) in olive fruit fly males^37^. One might argue that the use of a self-selection method is not ideal to study winner and loser effects^4^. However, we recently repeated these experiments on a closely related tephritid species, the Mediterranean fruit fly *Ceratitis capitata* (Wiedemann), using a random-selection procedure. In experiments testing both the winner and loser effect, we found that medflies experiencing two previous victories or defeats displayed higher aggression rates and achieved more victories in subsequent contests^57^.

Both in Experiments 1 and 2, we observed that olive fruit fly males displayed no consistent differences in intensity of aggression, fighting duration or success rates in relation to the two pre-experimental isolation periods. This indicates that prolonged social isolation does not magnify the expression of aggressive behaviour in *B. oleae* males. In some animal species individuals isolated for long periods of time are more aggressive than non-isolated ones, while in other species it is overcrowding that is positively correlated with aggression^1,26,58^. It can be argued that the higher levels of aggression and longer contest durations found in twofold winner—naïve and twofold loser-naïve fighting pairs of *B. oleae* do not give precise information on the aggressive behaviour of each fly in a contest^29^. However, the aggressive behaviour of naïve males tested in our experiments is assumed to be constant within *B. oleae*, thus it appears that the differences observed in intensity of aggression and duration of contest are due to the previous victories or defeats experienced by winner and loser flies, respectively. Further studies are needed to quantify the fighting performances of naïve and winner/loser males at the individual level.

In Experiment 3, we evaluated if winning and losing probabilities are affected solely by the outcome of the previous contests, or if they required actual experience in physical combat. Results demonstrated that the enhanced fighting performance of both winner and loser males was influenced by merely experiencing a previous contest, regardless of the outcome or even the occurrence of physical contact in previous contests. In the olive fruit fly, the experience of winning without physical combat evoked a behavioural effect similar to that detected in crickets, as in both species this experience alone is sufficient to enhance aggression and prolong fight duration in subsequent male-male contests^10^. This has an interesting parallel in humans, as it has been demonstrated that watching a previous victory raises the level of the aggression-promoting hormone testosterone^59^. Conversely, in other vertebrate species (e.g. the East African cichlid fish), it has been shown that fighting experience itself (coupled with an androgen response) increases the subsequent likelihood of winning, even in the absence of a prior winning experience^60,61^. We demonstrated that experiencing two consecutive defeats without physical contact induced a similar effect in olive fruit flies males (losers without fighting), who achieved higher fighting success in subsequent combat, at a level comparable to males that had experienced two consecutive defeats via physical combat (losers with fighting). Moreover, the fighting success of “losers with fighting” and “losers without fighting” is comparable to “winners with fighting” and “winners without fighting”, indicating that experiencing either consecutive victories or defeats, with or without physical contests, evoked enhanced aggression levels in the olive fruit fly and enhanced male fighting success in future contests (see also Stamps and Krishnan^54^).

Overall, although extensive research has been carried out to understand how social experiences affect the outcomes of contests in animals, the ultimate and proximate causes for the existence of the winner and loser effects are still unknown^4,26,32^. Rutte et al.^32^ formulated two adaptive hypotheses to explain these effects, namely the “social-cue hypothesis” (i.e. victory and defeat leave traces that affect the decisions of subsequent opponents) and the “self-assessment hypothesis” (i.e. winners and losers gain information about their own relative fighting ability in the population). Our findings provide evidence that olive fruit fly males that experienced previous defeats escalated to higher levels of aggression, fought longer in subsequent contests and achieved higher fighting success, allowing us to hypothesize that natural selection has operated in ways that favour animals increasing aggression in consecutive contests, regardless of past outcomes. Further research is needed on the neuroendocrine mechanisms mediating this unexpected experience-induced effect in tephritid contests, as well as on how these flies avoid protracted contests, assessing their decisions on the basis of differences in their resource-holding potentials (RHP) or through alternative strategies, such as “own RHP-dependent persistence” mechanisms”^22,62^.

## Methods

### Ethics statement

This research adheres to the ASAB/ABS Guidelines for the Use of Animals in Research (2012)^63^. All treatments of the experimental animals (*C. capitata*) complied with the laws of the country (Italy) in which it was performed (D.M. 116192) and the European Union regulations. All experimental procedures were approved by the University of Pisa Ethical Committee. No permits were needed from the Italian government for experiments involving *C. capitata*. All the experiments were based on behavioural observations. Flies were treated as well as possible given the constraints of the experimental design. None of them has been damaged or killed during the experiments.

### Insect rearing

Insects used in this study were obtained from pupae collected during January - February 2014. Olive fruit fly pupae were collected in a Tuscan olive-mill located in Pisa (Italy). A fine paintbrush was used to collect pupae from the bottom of boxes of olive fruits. The pupae were maintained under controlled conditions (21 ± 1°C, 55 ± 5% RH, 16:8 (L:D) photoperiod) in University of Pisa laboratories to wait for adult emergence^64^.

In all experiments, we tested both (*i*) males isolated for their entire life until the experimental phase and (*ii*) males exposed to artificially crowded conditions until 24 h before the experimental phase. In the first case, males were gently separated within 10 h of emergence and placed singly in clean Plexiglas cups (diameter: 40 mm, length: 7 mm), using a fine paintbrush. In the second case, within 10 h after emergence, males were stored in cylindrical Plexiglas cages (400 mm diameter, height 500 mm) at a density of 100 individuals per cage^64^. Then, 24 h before the experimental phase they were moved to the conditions described in (*i*). In both cases, olive fruit fly adults were fed a dry diet of yeast extract (Sigma-Aldrich) and sucrose mixture, at a ratio of 1:10 (w:w). Water was provided separately on a cotton wick.

### General observations

Experiments were conducted in March - April 2014 in a laboratory room illuminated with fluorescent daylight tubes [16:8 (L:D) photoperiod, lights on at 06:00]. Neon tubes (Philips 30 W/33) provided light intensity in close proximity of the testing arena of approximately 1,000 lux, estimated over the 300–1,100 nm waveband using a LI-1800 spectroradiometer (LI-COR Inc., Lincoln, NE, USA), equipped with a remote cosine receptor. Directional light cues were avoided by using diffused laboratory lighting to reduce possible reflection and phototaxis. Experiments were performed in a Plexiglas testing arena (diameter: 150 mm; length: 200 mm). A fly entrance hole (diameter: 10 mm) was made on the top, in the central part of the arena. Both ends of the arena were covered with transparent chiffon fabric (mesh size: 0.05 mm). The arena contained a twig of olive, *Olea europea* L. cultivar “Frantoio”, with ten leaves. The olive twig and the chiffon fabrics used at the ends of the arena were changed for each replicate. After each replicate, the arena was carefully washed for about 30 s with warm water at 35–40°C, then cleaned using water plus mild soap for about 5 min, then rinsed with hot water for about 30 s and finally rinsed with distilled water at room temperature^65^.

Virgin males (age: 12–20 days old) were used in all experiments. For each replicate, flies were replaced by new ones of the same age. Twenty-four hours before the testing phase, all categories of males were cooled for 3 min at −10°C, marked with a small dot of nontoxic colour paint (Polycolor, Maimeri, Italy) on the thorax and weighed. Only flies with a body mass ranging from 4–4.5 mg were tested^21^. Preliminary experiments and previous research^21^ showed that cooling and colour tagging did not influence fly behaviour. Experiments were performed over 60 days to account for any daily variability. All experiments were carried out from 10:00–17:00 h. Only interactions in which the winning male remained on the territory for at least 30 s after the conclusion of the aggressive interaction were considered for data analysis^21^.

Since the winner and loser effects in invertebrates are transient^28^, we tested two different inter-fight intervals (5 and 15 min^28^). Further, it has been noted that social isolation is a major factor affecting the intensity and outcome of aggressive behaviour in invertebrates^26^, although a number of studies on aggression were conducted with animals maintained in artificially crowded conditions until 24 h before the experimental phase^4,28^. We tested, in all experiments, both animals exposed to artificially crowded conditions and individuals isolated until the experimental phase.

### Detecting experience effects: self-selection *versus* random selection

To evaluate the effects of winning (Experiment 1) and losing (Experiment 2), we staged aggressive interactions between pairs of male flies, using the knockout tournament methods described by Rillich and Stevenson^29^, with slight modifications. One could argue that self-selection is not ideal for measuring winner and loser effects, since with this approach the particular winning/losing experience cannot be disentangled from intrinsic differences in fighting ability^4^. A preferable method is the random-selection procedure, in which focal individuals are randomly allocated to experimental groups and pitted against either a much stronger or weaker opponent, to deliver the winning or loser experience^4^. Unfortunately, the latter method is not applicable to the majority of tephritid flies, since true predictors^31,36,37,66^ of fighting outcomes are not available for these insects^45,46^, with the exception of oriental fruit fly females^67^ and males of a gall-forming fly, *Procecidochares* sp.^68^. However, the self-selection procedure has also some merits. First, self-selection appears be a more “natural” method for contestants to acquire experiences, over the random-selection one^4^. Second, a number of studies have been conducted to investigated winner and loser effects on insect species (e.g. crickets and ants) and led to similar results^4,28,51^, showing that the effect of intrinsic fighting ability is not a major factor that conflate the effect of previous fighting experience. Third, Bégin et al.^69^ reported that self-selected winners have a 0.67 probability of having intrinsically higher fighting ability than a size-matched naïve opponent. This would suggest testing a null hypothesis of 0.67 for self-selected winners and 0.33 for self-selected losers. This is not possible in this study, due to the simultaneous presence of both winners and losers in Experiment 3. However, concerning the loser effect, our main result higher aggression levels and higher fighting success in self-selected losers over naïve flies. This is a further demonstration of the reliability of our results: even if self-selected losers have a 0.33 probability of having an intrinsically higher fighting ability than naïve opponents, testing a null hypothesis of 0.5^69^, we still found fighting-induced hyper-aggression in the olive fruit fly.

### Experiment 1: the winner effect

To evaluate the effects of winning we staged aggressive interactions between pairs of male olive fruit flies, using the methods described by Rillich and Stevenson^29^, with slight modifications. Two males with no previous fighting experience (naïve males) were first matched against each other. Each contestant was gently transferred onto the floor of the cylindrical arena using a clean glass vial and observed for 60 min. They usually started to explore the olive twig. When males came in close proximity to one another, a fly started wing waving acts, the first level of escalating aggression that characterizes male-male contests (Table 1)^21,45^. Head butting and boxing acts can follow wing-waving motions^21,45^. The winner of the first round (winner) was then matched against a new naïve contestant. The male that won twice in succession (twofold winner) was matched against a third naïve male in the final round. All twofold winners experimented two previous consecutive victories. All twofold winners were previously tested as winners. All winners were previously tested as naïve males. The inter-fight interval between consecutive fights was 5 min or 15 min (Table 2).

**Table 1:** Escalating level of aggression that characterizes male-male contests in the olive fruit fly, *Bactrocera oleae*

Level	Behavior	Description
0	Avoidance (both)	Mutual avoidance: non-aggressive interaction
1	Avoidance (one)	Pre-established dominance: one male attacks, the other retreats
2	Wing waving (one)	Attacker faces the opponent and brings both wings forward perpendicular to the longitudinal axis of its body, while ventral surface of wings are turned to face anterior
3	Wing waving (both)	Both males perform wing waving acts
4	Chasing	Running towards the opponent
5	Pouncing	Lunging at the opponent ending with head butting
6	Boxing (one)	Attacker raises forelegs, repeatedly and alternately hitting opponent on the head and thorax
7	Boxing (both)	Both males grasp each other with forelegs

**Table 2:**
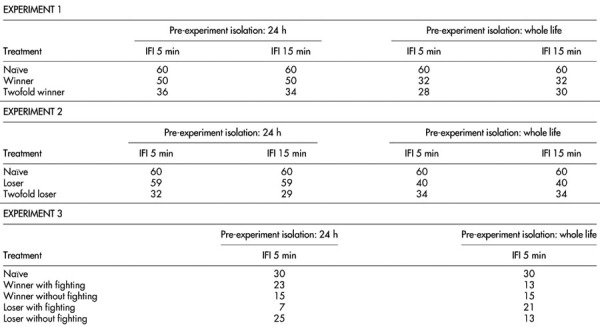
Tested flies for each experiment. Naïve = fight-inexperienced male. Winner = winner of one previous encounter. Twofold winner = winner of two previous encounters. Loser = loser of one previous encounter. Twofold loser = loser of two previous encounters. IFI = inter-fight interval between training and testing phase. Winners with fighting = winner male that had won twice in succession with physical fighting. Winner without fighting = winner male that had won twice in succession without physical fighting. Loser with fighting = loser male that had lost twice in succession with physical fighting. Loser without fighting = loser male that lost twice in succession without physical fighting

For each replicate, the following parameters characterizing aggressive behaviour were recorded: (*a*) the intensity of aggression, scored from 0 to 7, in agreement with the escalating aggression that characterizes male-male contests in the olive fruit fly^21^, reported in Table 1; (*b*) the duration of the entire contest (s); (*c*) the outcome of the contest, i.e. which male was dislodged from the leaf at the end of the aggressive interaction^21^. The number of observations for each treatment is provided in Table 2.

### Experiment 2: the loser effect

To evaluate the effects of losing we staged contests between pairs of male olive fruit flies, using the methods described above for the winner effect. Individuals tested here were a completely separate subset from Experiment 1. Two males with no previous fighting experience were first matched against each other. Each contestant was gently transferred onto the floor of the cylindrical arena using a clean glass vial and observed for 60 min. The loser of the first round (loser) was then matched against a new naïve contestant. The male that lost twice in succession (twofold loser) was matched against a third naïve male in the final round. All twofold losers experienced two consecutive previous defeats. All twofold losers were previously tested as losers. All losers were previously tested as naïve males. The inter-fight interval between consecutive fights was 5 min or 15 min (Table 2).

For each replicate, the following parameters characterizing the aggressive behaviour were recorded: (*a*) the intensity of aggression (Table 1); (*b*) the duration of the entire contest (s); (*c*) the outcome of the contest. The number of observations for each treatment is provided in Table 2.

### Experiment 3: role of physical combat in determining winner and loser effect

To test whether the winner and loser effects depend solely on experiencing physical combat with a contestant, we staged contests using the methods described in Experiments 1 and 2. Individuals tested here were a completely separate subset from Experiments 1 and 2. We observed aggression initially between naïve pairs of male olive fruit flies for two consecutive bouts, obtaining four categories of males: (*1*) males that won twice in succession with fighting (winners with fighting); (*2*) males that won twice in succession without fighting (winners without fighting); (*3*) males that lost twice in succession with fighting (losers with fighting); (*4*) males that lost twice in succession without fighting (losers without fighting) (i.e. “with fighting” = physical contact occurred between flies; “without fighting” = the contest reached wing waving as a maximum and did not involve physical contact). Naïve males were also tested as a control. The inter-fight interval between consecutive fights was 5 min for all tournaments. After an inter-fight interval of 5 min, the males belonging to the five treatments described above were tested in the same experimental conditions against naïve males.

For each replicate, the following parameters characterising the aggressive behaviour were recorded: (*a*) the intensity of aggression (Table 1); (*b*) the duration of the entire fight; (*c*) the outcome of the fight. The number of observations for each treatment is provided in Table 2.

### Data analysis

In Experiments 1 and 2, aggression intensity and fighting duration data were analyzed using a General Linear Mixed Model (GLMM) (JMP SAS, 1999) with three factors: pre-experiment isolation, inter-fight interval and previously experienced fighting outcome: y_ijzw_ = μ + IS_i_ + IFI_j_ + EXP_z_ + IS_i_*IFI_j_ + IS_i_*EXP_z_ + IFI_j_*EXP_z_ + IS_i_*IFI_j_*EXP_z_ + ID_w_ + e_ijzw_, in which y_ijzw_ is the observation, μ is the overall mean, IS_i_ is the i-th fixed effect of pre-experiment isolation (i = 1–2; i.e. 24 h or lifetime), IFI_j_ is the j-th fixed effect of inter-fight interval (j = 1–2; i.e. 5 or 15 min), EXP_z_ is z-th fixed effect of the previously experienced fighting outcome (j = 1–3; i.e. naïve, winner or twofold winner for Experiment 1; naïve, loser or twofold loser for Experiment 2), ID_w_ is the w-th random effect of the individual over repeated testing phases (W = 1–60) and e_ijzw_ the residual error. Averages were separated by the Tukey's HSD test. A probability level of *P* < 0.05 was used to test significance of differences between means. Differences in fighting success among different treatments were evaluated using the GLMM described above with a binomial error structure (to model win/loss outcomes) and male ID as a random effect (α = 0.05).

In Experiment 3, aggression intensity in male-male contests and fight duration data were analyzed using a GLMM with two factors and binomial error structure: pre-experiment isolation and previously experienced fighting outcome: y_ijw_ = μ + IS_i_ + EXP_j_ + IS_j_*EXP_j_ + ID_w_ + e_j_, in which y_j_ is the observation, μ is the overall mean, IS_i_ is the i-th fixed effect of pre-experiment isolation (j = 1–2; i.e. 24 h or lifetime), EXP_j_ is the j-th fixed effect of previously experienced fighting outcome (j = 1–5), ID_w_ is the w-th random effect of the individual over repeated testing phases (w = 1–60) and e_j_ the residual error. Averages were separated by the Tukey's HSD test. A probability level of *P* < 0.05 was used to test significance of differences between means. Differences in fighting success among different treatments were evaluated using the GLMM described above with a binomial error structure (to model win/loss outcomes) and male ID as a random effect (α = 0.05).
